# Supplementary material for: Allosteric disulfide control of ligand binding and endocytosis of KIR2DL4, the natural killer cell receptor for HLA-G
Source: bioRxiv. 2026 Jul 10:2026.02.18.706601. Preprint. [Version 2] doi: 10.64898/2026.02.18.706601 (PMC13371052; doi:10.64898/2026.02.18.706601)
Supplement: Supplement 1 [file NIHPP2026.02.18.706601v2-supplement-1.pdf]

bioRxiv preprint doi: https://doi.org/10.64898/2026.02.18.706601; this version posted July 10, 2026. The copyright holder for this preprint (which was not certified by peer review) is the author/funder. This article is a US Government work. It is not subject to copyright under 17 USC 105 and is also made available for use under a CC0 license.

Supplementary information for:

Allosteric disulfide control of ligand binding and endocytosis of the natural killer cell receptor for HLA-G

Sumati Rajagopalan, Joyce Chiu, Priyanka Chaurasia, Jinghua Lu, George M. Mastorakos, Saurav Majumder, Kristof Nolan, Jan Petersen, Erin J. Adams, Peter Sun, Jamie Rossjohn, Philip J. Hogg and Eric O. Long

Corresponding author: Eric O. Long

Email: eLong@nih.gov

This file includes

Figures S1 to S7

Tables S1 to S2

Other supporting materials for this manuscript include the following:

Dataset S1

bioRxiv preprint doi: https://doi.org/10.64898/2026.02.18.706601; this version posted July 10, 2026. The copyright holder for this preprint (which was not certified by peer review) is the author/funder. This article is a US Government work. It is not subject to copyright under 17 USC 105 and is also made available for use under a CC0 license.

Fig.S1

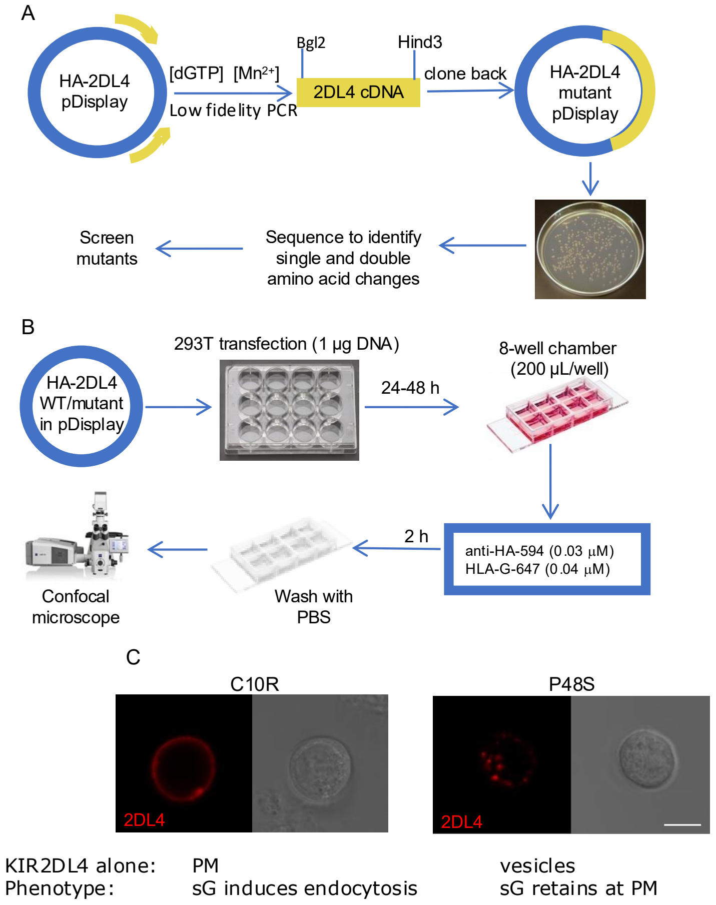

D

HLA-G:	none or very low		vesicles		PM
KIR2DL4:	vesicles	PM	vesicles	PM and vesicles	PM
KIR2DL4 without sG:	vesicles	PM	vesicles	PM	vesicles
Phenotype:	–	no endocytosis	WT	sG-induced endocytosis	no endocytosis with sG
Targeted mutants:	3	0		4	2
Examples:	C74R	–		C10S	F176A

bioRxiv preprint doi: https://doi.org/10.64898/2026.02.18.706601; this version posted July 10, 2026. The copyright holder for this preprint (which was not certified by peer review) is the author/funder. This article is a US Government work. It is not subject to copyright under 17 USC 105 and is also made available for use under a CC0 license.

Fig. S1: Random mutagenesis screen

(**A**) Strategy for the generation of mutants. (**B**) Examination of mutants by confocal microscopy. WT = wild-type. (**C**) In the same assay as in Figure 1C, mutants C10R and P48S were tested in the absence of soluble HLA-G (sG). Confocal images of anti-HA-Alexa-594 revealed their location. A DIC image is shown in the right panel. PM = plasma membrane. (**D**) Flow chart of mutants distributed among 4 categories distinct from WT. Several mutants from each category were also tested in the absence of soluble HLA-G (sG). HLA-G often dictates the location of KIR2DL4. For example, C10R internalization depended on HLA-G, and P48S was not internalized into vesicles in the presence of soluble HLA-G.

bioRxiv preprint doi: https://doi.org/10.64898/2026.02.18.706601; this version posted July 10, 2026. The copyright holder for this preprint (which was not certified by peer review) is the author/funder. This article is a US Government work. It is not subject to copyright under 17 USC 105 and is also made available for use under a CC0 license.

Fig. S2

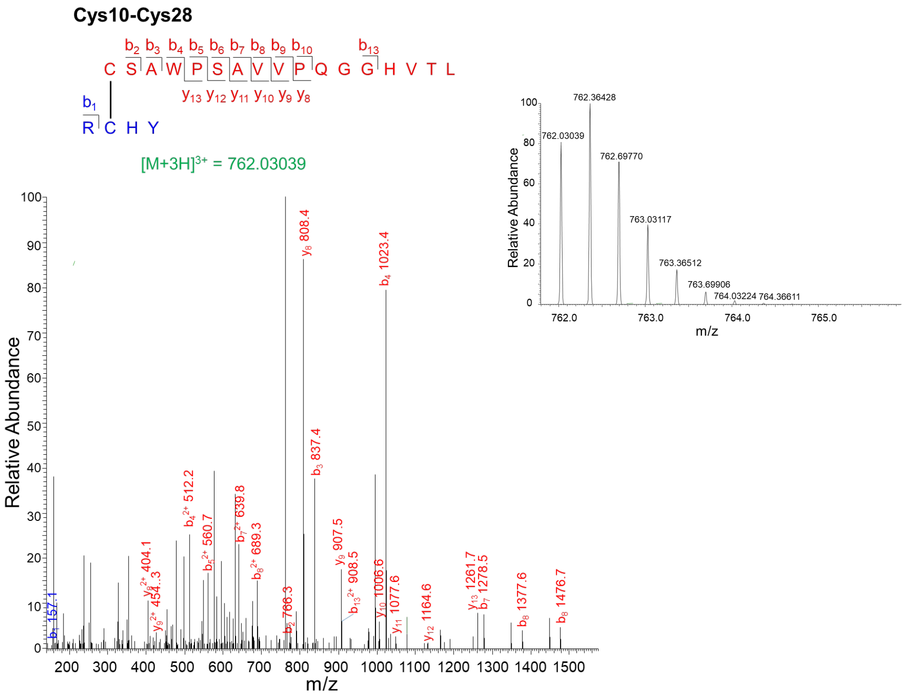

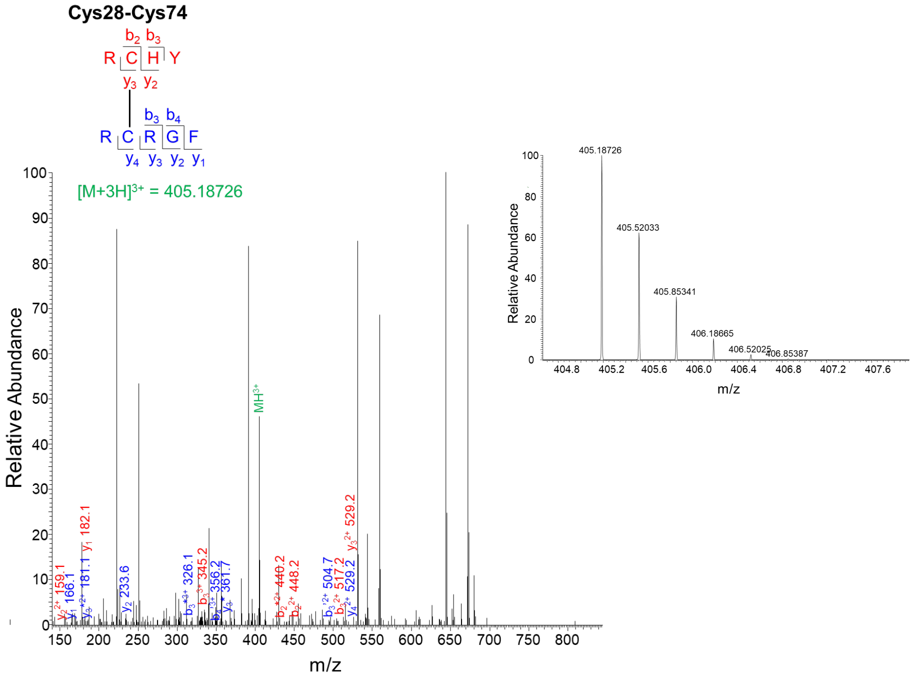

bioRxiv preprint doi: https://doi.org/10.64898/2026.02.18.706601; this version posted July 10, 2026. The copyright holder for this preprint (which was not certified by peer review) is the author/funder. This article is a US Government work. It is not subject to copyright under 17 USC 105 and is also made available for use under a CC0 license.

Fig. S2: Analysis of disulfide-linked peptides in recombinant KIR2DL4 protein by mass spectrometry.

Tandem mass spectra of the peptides linked by disulfide bond between Cys10 and Cys28 and between Cys28 and Cys74 are shown. The accurate mass of Cys10-Cys28 and Cys28-C74 peptides are shown in the insets (Cys10-Cys28: [M + 3H]3+ = *m*/*z* 762.03039 and expected [M + 3H]3+ = *m*/*z* 762.0299; Cys28-Cys74 : [M + 3H]3+ = *m*/*z* 405.18726 and expected [M + 3H]3+ = *m*/*z* 405.1870).

bioRxiv preprint doi: https://doi.org/10.64898/2026.02.18.706601; this version posted July 10, 2026. The copyright holder for this preprint (which was not certified by peer review) is the author/funder. This article is a US Government work. It is not subject to copyright under 17 USC 105 and is also made available for use under a CC0 license.

Fig.S3

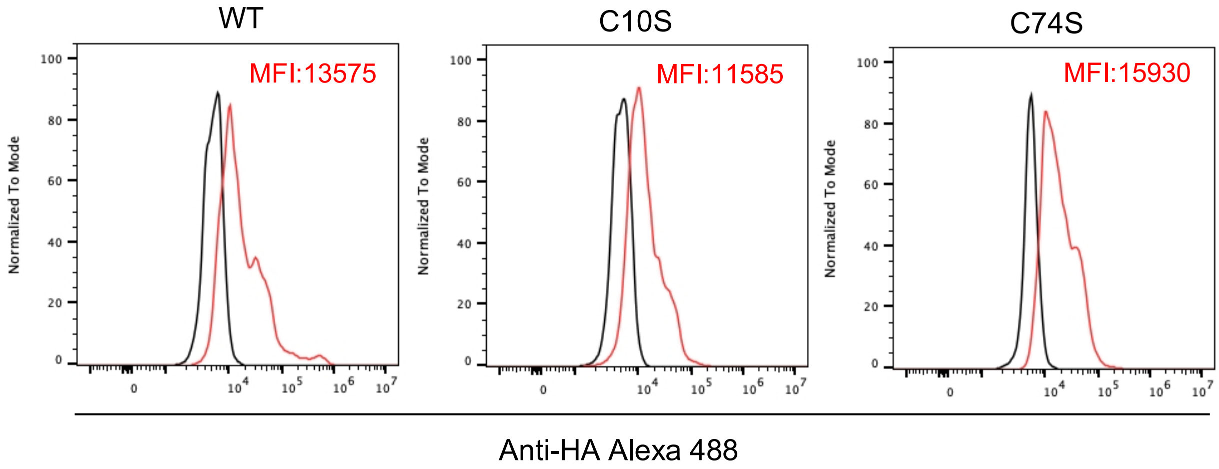

Fig. S3: Flow cytometry profiles of 293T cells stably expressing WT, C10S or C74S mutants of HA-tagged KIR2DL4. Cells were stained with anti-HA tag antibody coupled to Alexa 488. Median fluorescence intensity (MFI) for the red profiles is indicated in the upper right corner.

bioRxiv preprint doi: https://doi.org/10.64898/2026.02.18.706601; this version posted July 10, 2026. The copyright holder for this preprint (which was not certified by peer review) is the author/funder. This article is a US Government work. It is not subject to copyright under 17 USC 105 and is also made available for use under a CC0 license.

Fig. S4

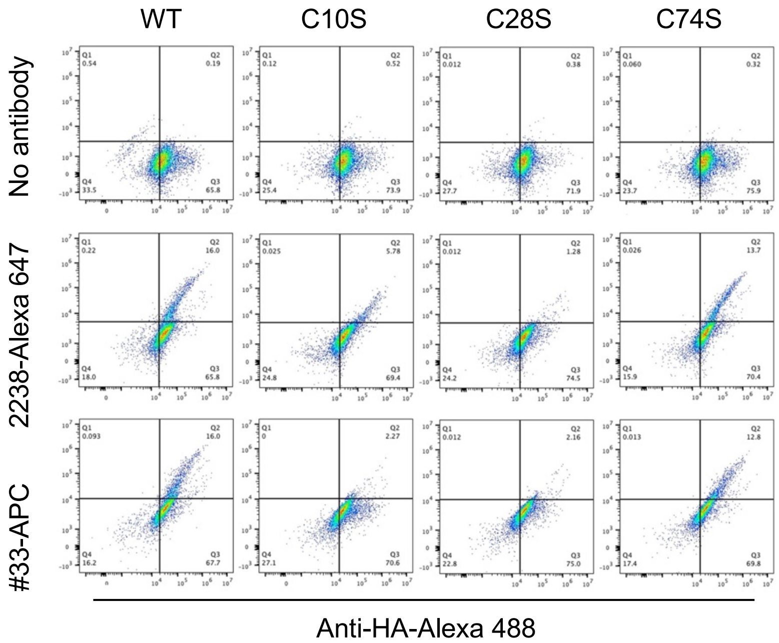

Fig. S4

Flow cytometry profiles of 293T cells expressing HA-tagged KIR2DL4 WT and, in parallel, the 3 Cys to Ser mutants loaded for 2 h at 37°C with an antibody to the HA-tag and two different antibodies to KIR2DL4. Staining with the control anti-HA antibody coupled with Alexa 488 is shown on the x axis versus no antibody (*top*), whereas mAb 2238-Alexa 647 (*center*) and mAb #33-APC (*bottom*) are on the y axis. The lower left quadrant was set to include 98% of unstained control cells.

bioRxiv preprint doi: https://doi.org/10.64898/2026.02.18.706601; this version posted July 10, 2026. The copyright holder for this preprint (which was not certified by peer review) is the author/funder. This article is a US Government work. It is not subject to copyright under 17 USC 105 and is also made available for use under a CC0 license.

Fig.S5

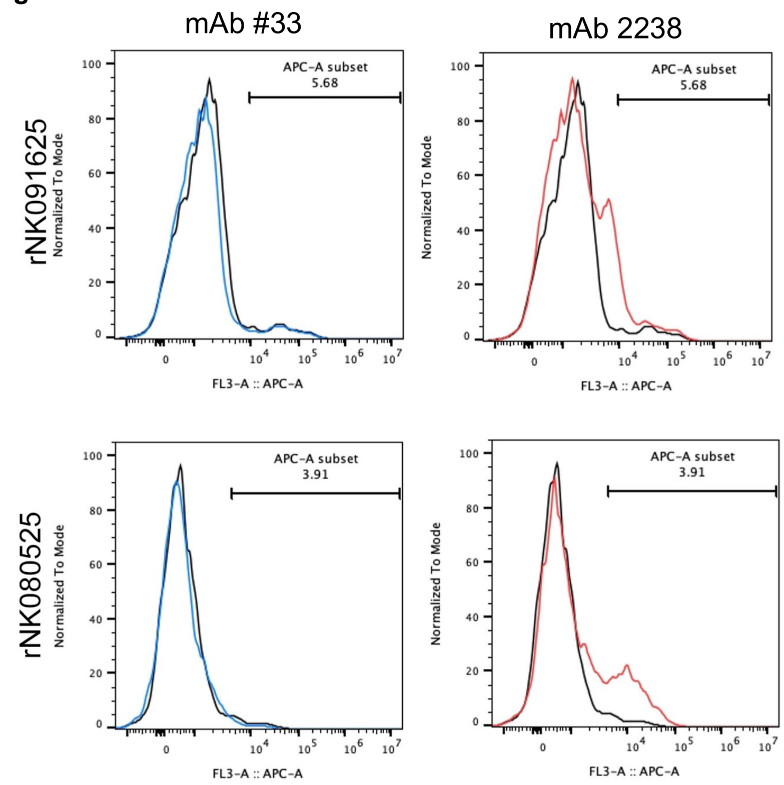

Fig.S5

Flow cytometry profiles of primary NK cells from two different donors stained at the cell surface with mAb #33 (blue profiles) or mAb 2238 (red profiles) followed by APC-coupled secondary antibodies. Control staining with secondary antibodies alone is also shown (black profiles).

bioRxiv preprint doi: https://doi.org/10.64898/2026.02.18.706601; this version posted July 10, 2026. The copyright holder for this preprint (which was not certified by peer review) is the author/funder. This article is a US Government work. It is not subject to copyright under 17 USC 105 and is also made available for use under a CC0 license.

1094

1095 **Fig.S6**

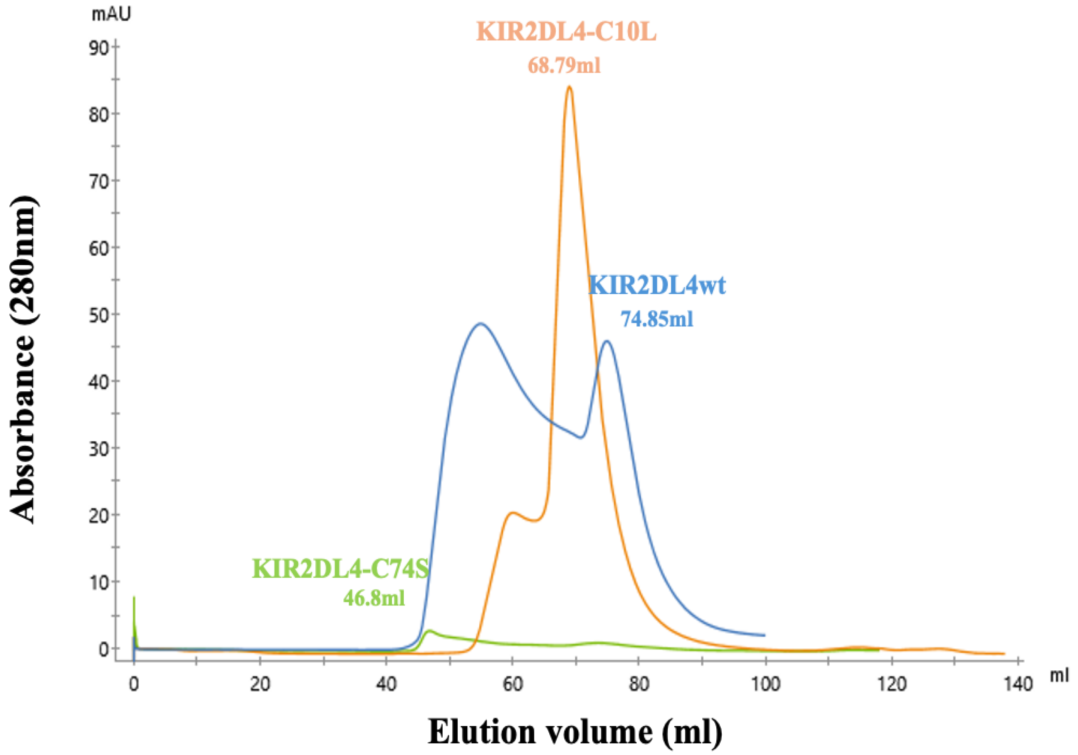

1096

1097 **Fig.S6**

1098 Size exclusion chromatography (SEC) profiles. Overlay of size exclusion profile with

1099 elution volume of purified KIR2DL4 wt (blue), KIR2DL4 C10L (orange) and KIR2DL4

1100 C74S (green).

1101

1102

bioRxiv preprint doi: https://doi.org/10.64898/2026.02.18.706601; this version posted July 10, 2026. The copyright holder for this preprint (which was not certified by peer review) is the author/funder. This article is a US Government work. It is not subject to copyright under 17 USC 105 and is also made available for use under a CC0 license.

Fig.S7

A **B**

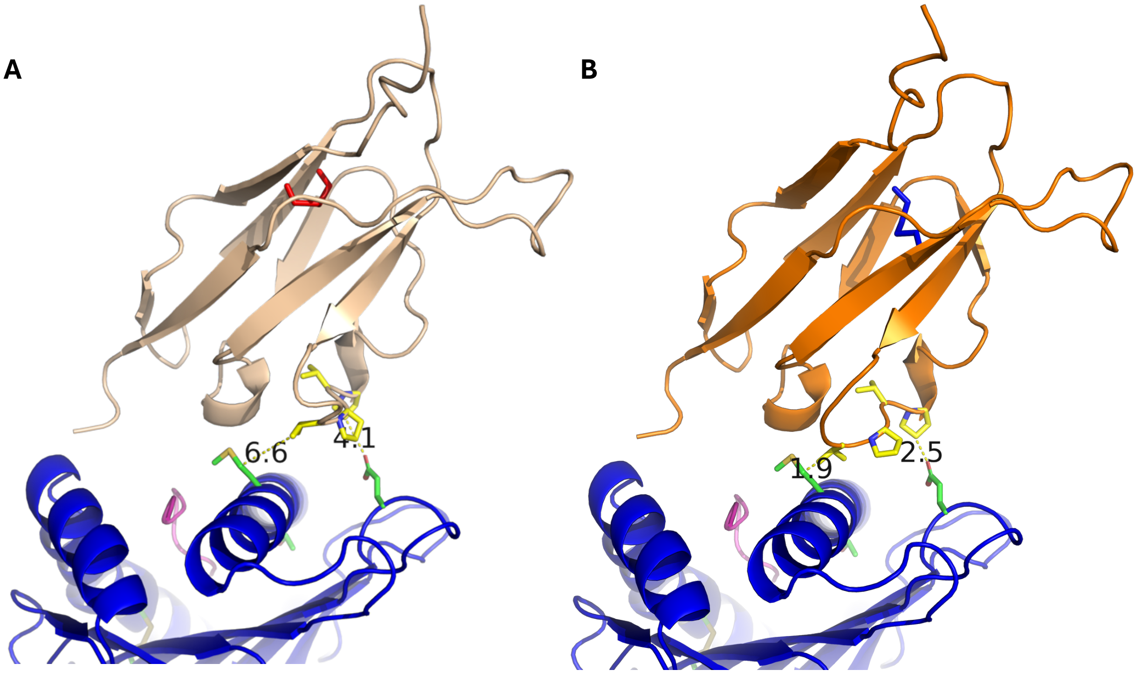

C74R (beige); HLA-G (blue) C10R (orange); HLA-G (blue)

C

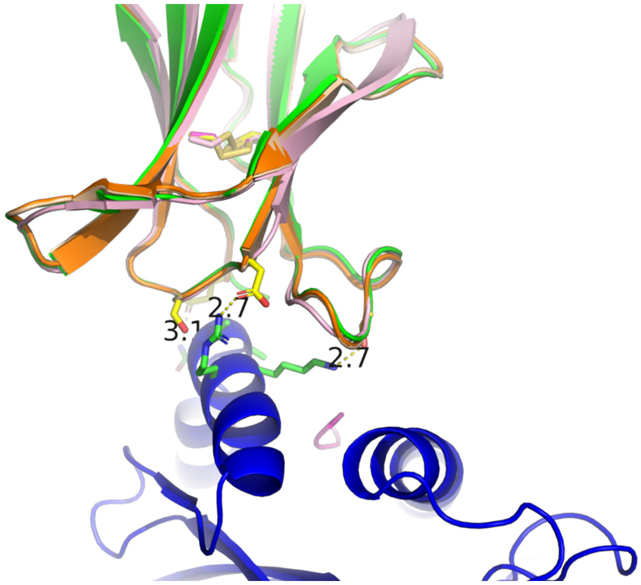

KIR2DL4: WT 3WYR (pink) WT AF3 (green) C74R (beige) C10R (orange) HLA-G (blue)

bioRxiv preprint doi: https://doi.org/10.64898/2026.02.18.706601; this version posted July 10, 2026. The copyright holder for this preprint (which was not certified by peer review) is the author/funder. This article is a US Government work. It is not subject to copyright under 17 USC 105 and is also made available for use under a CC0 license.

Fig. S7. Potential interaction of KIR2DL4 with HLA-G, as predicted by AlphaFold

(**A**) Docking of the C74R KIR2DL4 (*beige*) with HLA-G (*blue*), as predicted by AlphaFold 3. The structure of KIR2DL4 in this Cys10-Cys28 configuration is similar to that of the crystal structure (Fig. 7C). (**B**) C10R KIR2DL4 (*orange*) with HLA-G (*blue*). The predicted structure of this Cys28-Cys74 KIR2DL4, with the Val45-Pro48 loop closer to HLA-G, resembles that of WT KIR2DL4 (Fig. 7D). (**C**) Predicted folding of the D2 domain in the four KIR2DL4 isoforms (3WYR crystal structure of WT, and AlphaFold predictions of WT, C74R, and C10R) examined here, indicate a conserved D2 domain folding (shown as an overlay). Several predicted contacts of KIR2DL4 with HLA-G (*blue*) are similar to those of KIR3DL1 D2 domain with HLA-B, such as Ser128–Arg145 (3.1 Å, *left*), Asp130–Arg145 (2.7 Å, *center*), and Ser179–Lys146 (2.7 Å, *right*). As KIR2DL4 lacks the 100 amino acid-long D1 domain of KIR3DL1, the Ser128, Asp130, and Ser179 of KIR2DL4 correspond to Ser228, Asp230, and Ser279 of KIR3DL1.

bioRxiv preprint doi: https://doi.org/10.64898/2026.02.18.706601; this version posted July 10, 2026. The copyright holder for this preprint (which was not certified by peer review) is the author/funder. This article is a US Government work. It is not subject to copyright under 17 USC 105 and is also made available for use under a CC0 license.

Table S1

Table S1 KIR2DL4 peptides analysed to determine disulfide bond redox state. Peptides were detected by Byonic analysis software, confirmed by MS/MS and have errors <6 ppm. Only peptides with peak areas >10 million for a given Cys were included in the analysis.

Cys	Peptide sequence*	Peptide score#	Peptide error (ppm)
10	**C**SAWPSAVVPQGGHVTL	549.9	0.16
	**C**SAWPSAVVPQGGH	403.3	0.8
	**C**SAW	351.1	0.8
28	R**C**HY	255.5	0.3
74	R**C**RGF	245.7	0.72
123	S**C**SSQSSF	383.9	0.97
	S**C**SSQSSFDIY	387.2	0.32
172	R**C**FGSF	367.9	0.91

*Cys was labelled with ^{12}C-iodoacetanilide or ^{13}C-iodoacetanilide and is in bold.
#Peptide score calculated by Byonic analysis software is a primary indicator of peptide-spectrum matches.

bioRxiv preprint doi: https://doi.org/10.64898/2026.02.18.706601; this version posted July 10, 2026. The copyright holder for this preprint (which was not certified by peer review) is the author/funder. This article is a US Government work. It is not subject to copyright under 17 USC 105 and is also made available for use under a CC0 license.

Table S2 Summary of SPR measurements

**Epitope**	**HLA**	**KIRs**	**K_D (μM)**
Pp65-2-VLPHETRLL	HLA-G wt	KIR2DL4*001	19.4 μM ± 4.00
Pp65-2-VLPHETRLL	HLA-G wt	KIR2DL4*001C10L	1.59 ± 0.08
Pp65-2-VLPHETRLL	HLA-GC42S	KIR2DL4*001C10L	1.65 ± 0.07
Pp65-2-VLPHETRLL	HLA-G wt	KIR3DL1*001	>300
Pp65-2-VLPHETRLL	HLA-GC42S	KIR3DL1*001	>300
H2A-RIIPRHLQL	HLA-G wt	KIR2DL4*001	N.B.
Pp65-6-VFPTKDVAL	HLA-G wt	KIR2DL4*001	N.B.
H2A-RIIPRHLQL	HLA-G wt	KIR2DL4*001C10L	N.B.
Pp65-6-VFPTKDVAL	HLA-G wt	KIR2DL4*001C10L	N.B.
H2A-RIIPRHLQL	HLA-GC42S	KIR2DL4*001C10L	N.B.
Pp65-6-VFPTKDVAL	HLA-GC42S	KIR2DL4*001C10L	N.B.

KIR binding to the immobilised HLA-G epitopes was measured in two independent experiments. K_D was calculated from all data using one site-specific binding model. N.B. No binding.
